# Amelogenesis imperfecta. case report

**DOI:** 10.21142/2523-2754-1102-2023-156

**Published:** 2023-06-29

**Authors:** Nicole Alessandra Herrera-Rojas, Guido Alberto Perona-Miguel de Priego

**Affiliations:** 1 Pediatric Dentistry Department, Cayetano Heredia Peruvian University. Lima, Peru. nicole.herrera.r@upch.pe, guido.perona@upch.pe Universidad Peruana Cayetano Heredia Pediatric Dentistry Department Cayetano Heredia Peruvian University Lima Peru nicole.herrera.r@upch.pe guido.perona@upch.pe

**Keywords:** amelogenesis imperfecta, tooth, dental enamel, pediatric dentistry, amelogénesis imperfecta, diente, esmalte dental, odontopediatría

## Abstract

The main origin of amelogenesis imperfecta (AI) is a genetic alteration inherited by a family member which affects the dental enamel of the teeth of a person with this condition in various ways. The present clinical case from the Teaching Dental Clinic of the Peruvian University Cayetano Heredia is of a 6-year 5-month-old male child who came to the dental office accompanied by his father and 8-year-old sister, diagnosed with the same AI condition. The comprehensive treatment proposed for this patient was determined by radiographic and clinical examinations and consultations with specialists in different areas. The purpose of this publication was to report a case and describe possible clinical approaches.

## RESUMEN

El principal origen de la amelogénesis imperfecta (AI) es una alteración genética heredada por un familiar que afecta de diversas formas el esmalte dental de los dientes de una persona con esta afección. El presente caso clínico de la Clínica Odontológica Docente de la Universidad Peruana Cayetano Heredia se trata de un niño de sexo masculino de 6 años 5 meses que acude al consultorio odontológico acompañado de su padre y su hermana de 8 años, diagnosticados con la misma condición de AI. El tratamiento integral propuesto para este paciente estuvo determinado por exámenes radiográficos, clínicos y consultas con especialistas en diferentes áreas. El propósito de esta publicación fue reportar un caso y describir posibles enfoques clínicos.

## INTRODUCTION

Amelogenesis imperfecta (AI) is a disease mainly arising from an inherited genetic alteration ^1^, in which the dental enamel of both dentitions is affected. AI is classified into hypoplastic, hypomature, hypocalcified and hypoplastic - hypocalcified AI with taurodontism ^2^. In addition, variants of genes that affect AI influence enamel formation and provide new ways of pathogenesis ^3^. According to Aldred *et al*. ^4^ the criteria for the classification of AI include the phenotype, mode of inheritance, molecular defect and biochemical study, with the latter three being necessary to achieve a definitive diagnosis.

The presence of AI is a negative health burden for life if not timely diagnosed and treated, with accentuated sensitivity often leading to the avoidance of foods that cause pain and a consequent change in adequate eating habits as well as altered dental aesthetics, which can cause psychological distress ^5^^,^^6^. The purpose of this case report was to describe a pediatric case with AI and evaluate possible clinical approaches.

## CASE REPORT

**Figure 1:**
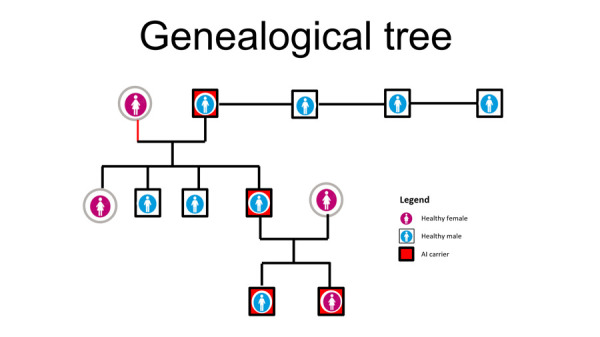
Genealogical Tree, representing two generations previous to the child affected.

**Figure 2:**
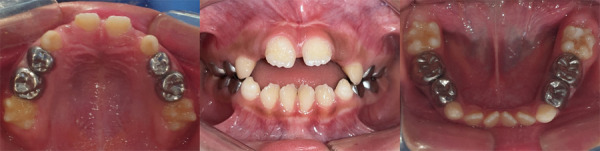
Occlusal and frontal photographs. Presents anterior open bite, deciduous canines and incisors, and permanent molars with enamel with a jagged and irregular appearance, metal crowns on all deciduous molars.

**Figure 3:**
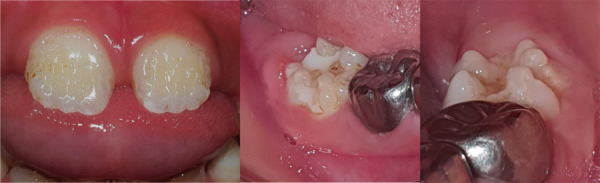
Enamel defect. Amelogenesis imperfecta with screening-type enamel, well determined by the contour of the teeth, generalized.

The parents of the patient gave their consent for the use of images and other clinical information from this case to be published. A 6-year 5-month-old male child was seen in the dental office of the Pediatric Dentistry Service of the Dental Clinic with his parents who reported that their son was sensitive to changes in temperature, especially cold in the upper anterior incisors. The father reported that the AI condition had been present in 3 generations with only 1 (male) person affected out of every 4 children in the first two generations and in the last generation 2 people had been affected out of 2 children (male and female) [Figure 1]. On extraoral examination, the patient presented facies of apparent mouth breathing, lip incompetence, dry lips, and a habit of tongue thrusting. On intraoral examination, he presented an anterior open bite, deciduous canines and incisors, and permanent molars with enamel with a sifted and irregular appearance, well determined by the contour of the teeth and metallic crowns on all deciduous molars. The oral hygiene of the patient was moderate due to the structure of the tooth surface and receptive behavior [Figure 2 and 3].

At 3 years of age, the patient was treated under general anesthesia for comprehensive restorative dental treatment. Subsequent controls were carried out, except during the last 2 and a half years due to the COVID-19 pandemic.

**Figure 4:**
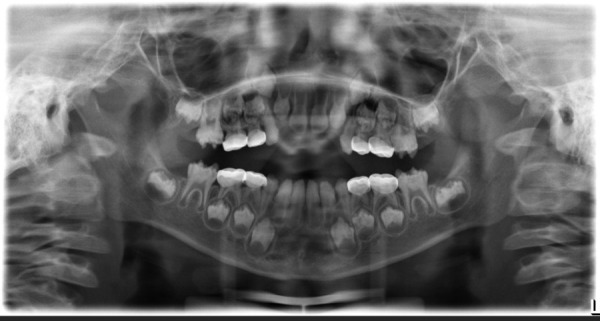
Panoramic radiography showing radiographic signs consistent with amelogenesis imperfecta. Misfitting preformed metal crowns alter the eruption of permanent molars.

In the panoramic radiograph seen in Figure 4, an alteration in the structure of the enamel is observed characterized by decreased density and irregularities of the occlusal surface of permanent teeth present in the mouth and in intraosseous evolution, being radiographic signs consistent with AI. Misadapted preformed metal crowns alter the eruption of the first permanent molars due to interference generated by the upper second deciduous molars [Figure 4].

Consultations with specialists in Otolaryngology were made due to the apparent mouth breathing presented by the patient and pediatricians were also consulted to assess the general status of the patient. Finally, Genetic Medicine was consulted to determine the definitive diagnosis of AI.

Dental treatment plan proposed:

• Behavior Management 

• Oral Hygiene Instruction 

• Dental prophylaxis + Application of Fluoride Varnish at 5%

• Direct restorations with composite resin.

• Change of preformed metal crowns in primary molars.

• Preformed metal crowns on permanent molars.

• Interconsultation with medical specialties.

**Figure 5:**
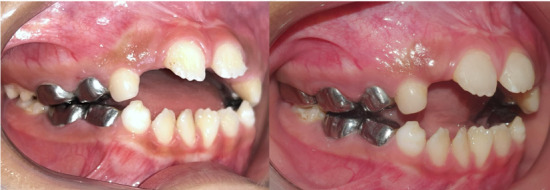
(a) Patient before treatment. (b) Patient after treatment.

Treatment progress [Figure 5]

• Tell-Show-Do

• Oral Hygiene Instruction Dental prophylaxis + Application of 5% Fluoride Varnish 3M® CLINPRO WHITE VARNISH

• Direct restorations of the upper anterior incisors, with 3M™ Filtek™ Z350 XT Color A Universal Restorative Composite, for the high sensitivity with respect to the anatomy characteristics of a patient of that age.

• Change of preformed metal crowns on primary molars 3M ESPE ® Stainless Steel Crown (Teeth N. 55, 54, 84, 85) eliminating the maladaptive anterior crowns, which interfere with the correct dental eruption process.

• Preformed metal crowns on permanent molars that have erupted and with little coronal structure due to a post-eruptive enamel fracture. (Teeth N. 16, 46)

• Change control of crowns and restorations to verify the integrity of the margins. Favorable evolution is observed and it is suggested to continue with the contralateral quadrants.

## DISCUSSION

The main challenge for a dentist in the case of enamel defects is correct diagnosis of the disease, which requires investigation of the family history and auxiliary tests. AI mainly occurs with a hereditary pattern and its diagnosis can be presumed by some previous family characteristics. However, in the case of adopted children, access to previous family history may be absent. In this scenario, clinical and radiographic characteristics are the only sources of information to achieve the diagnosis and make treatment decisions, and genotype-based methods should also ideally be performed ^6^^,^^7^.

The most common AI phenotype is type I, which accounts for 60-73% of all cases and is characterized by a deficiency in the amount of enamel, hypoplastic structure, reduced enamel thickness, and rough surface ^(8, 9)^. In the case of our patient, the characteristics of the enamel were generalized and well determined by the contour of the teeth, leading to a presumptive diagnosis of AI of the hypoplastic type. It was also known that this condition had been present in 3 generations, affecting both males and females of the family [Figure 1]. Hypoplastic AI with pits was suspected, being autosomal dominant according to Witkop ^10^, although a definitive diagnosis can only be achieved by determining molecular and biochemical defects.

A case report similar ^7^ to the present included a mother and her two children who manifested the disease. She was a 35-year-old woman with hypomature AI, while her eldest son (13 years old) had hypoplastic AI, and her youngest son (9 years old) had hypomature AI, demonstrating that the phenotypic manifestation of the disease had passed from the mother to both children. The key point to achieve good treatment is early diagnosis, evaluating all domains: function, activity, participation, personal and environmental. The later the diagnosis and care taken, the more additional disorders are established, complicating the treatment plan ^11^. In this sense, the collaboration of parents is important because AI requires a long-term treatment plan.

Improvement of the functional, aesthetic and masticatory component of the occlusal architecture is essential. Considering that among 60 children and young people between 5 and 17 years of age, more than 70% report that they sometimes experience pain or sensitivity and often feel unhappy with the appearance of their teeth ^3^^,^^12^. It is, therefore, important to achieve a comprehensive multidisciplinary treatment to minimize psychosocial difficulties due to the negative impact of AI, with preventive and restorative actions, and considering different behavior management techniques ^12^^-^^14^.

Our patient was first intervened at the age of 3, under general anesthesia, considering that he was not a receptive patient due to his age. The current treatment of the patient involves basic behavior management techniques with short and punctual appointments, prioritizing dental pieces presenting some symptomatology or clinical sign of inflammation and continuing with the treatments proposed according to the definitive diagnosis. The previous treatment was re-evaluated and interferences were found in the eruption of the first permanent molars due to bad adaptation of the crowns in the second deciduous molars. It was decided to place preformed crowns in the first molars in the process of eruption until the total dental replacement in order to preserve the dental structure until definitive restoration is possible.

Regarding treatment, any restorative decision is based on clinical characteristics such as pain, appearance and severity of the structural state of the piece and also according to the radiographic characteristics, choosing direct or indirect restorative treatments depending on the case ^11^.

Case reports of children describe direct composite restorations versus prefabricated composite veneers on anterior teeth and direct posterior composites in patients with AI, but more studies are needed in this regard ^11^^,^^15^. In the case of adults or complete permanent dentition, treatment is usually definitive, opting for fixed prostheses or the use of prefabricated veneers ^11^^,^^16^.

## CONCLUSION

Patients with AI usually present psychological conditions and functional alterations inherent to the disease, thereby making early treatment necessary to preserve the largest healthy dental structures and improve the prognosis of dental treatments. For deciduous dentition the treatments suggested are related to direct or temporary treatments that solve the functional problems of the patients, while for permanent teeth, definitive treatments can be considered with indirect and definitive techniques to restore function and aesthetics. In addition, multidisciplinary work is essential in this disease in order to manage all the problems related to this condition.
